# Contributing role of metabolic genes *APOE, FTO*, and *LPL* in the development of atrial fibrillation: insights from a case-control study

**DOI:** 10.1590/1806-9282.20240263

**Published:** 2024-08-16

**Authors:** Saira Rafaqat, Saima Sharif, Shagufta Naz, Dimitrios Patoulias, Aleksandra Klisic

**Affiliations:** 1 Lahore College for Women University, Department of Zoology - Lahore, Pakistan.; 2 Aristotle University of Thessaloniki, General Hospital "Hippokration", Second Department of Cardiology, Outpatient Department of Cardiometabolic Medicine - Thessaloniki, Greece.; 3 University of Montenegro, Faculty of Medicine - Podgorica, Montenegro.; 4 Primary Health Care Center, Center for Laboratory Diagnostics - Podgorica, Montenegro.

**Keywords:** Gene expression profile, Atrial fibrillation, Metabolic syndrome

## Abstract

**OBJECTIVE:**

The aim of the study was to examine the expression profile of genes (*APOE, FTO*, and *LPL*) associated with metabolic syndrome (MetS) in subjects with concomitant atrial fibrillation (AF).

**METHODS:**

A total of 690 subjects were categorized into control, AF without MetS, and AF with MetS.

**RESULTS:**

The expression profiles of the *APOE, FTO*, and *LPL* genes were decreased in AF subjects and AF subjects with MetS as compared to the controls. In AF without the MetS group, an inverse relationship was found between the expression of the *LPL* gene with body mass index (BMI) and a positive relationship with creatine kinase-MB, whereas expression of the *FTO* gene was inversely associated with fasting blood glucose and positively with cardiac troponin I in AF suffering from MetS. Expression of the *LPL* gene was directly linked with systolic blood pressure (SBP) and high-density lipoprotein-cholesterol (HDL-C), whereas an inverse correlation with heart rate and expression of the *FTO* gene in AF with MetS were shown. The expression of the *LPL* gene was inversely related to BMI in subjects with AF. The expression of the *LPL* gene was positively correlated with SBP and HDL-C and negatively correlated with heart rate, while the expression of the *FTO* gene was an important predictor of AF with MetS.

**CONCLUSION:**

The decreased expression of *APOE, FTO*, and *LPL* genes in AF with and without MetS indicates their potential contributing role in the pathogenesis of AF.

## INTRODUCTION

Atrial fibrillation (AF) is the most prevalent, sustained heart arrhythmia. Globally, more than 37 million people are affected by AF, which accounts for 0.51% of the world's population, while the prevalence of AF has increased by 33% over the past two decades. Future projections suggest that the absolute burden of AF could increase by more than 60% by the year 2050^1^.

Metabolic syndrome (MetS) diagnosis requires the presence of at least three out of five specific medical conditions, namely elevated fasting blood glucose (FBG), elevated blood pressure (BP) levels, elevated plasma levels of triglycerides (TG), low plasma levels of high-density lipoprotein cholesterol (HDL-C), and central obesity. The presence of MetS increases the risk of developing type 2 diabetes and cardiovascular disease^2^. It is well established that MetS and its components are linked to the development of AF^3,4^. It has been shown that the occurrence and development of AF may be influenced by a combination of various genes and/or environmental factors^5^.

The Apolipoprotein E (*APOE*) gene is situated on chromosome 19q13 and is responsible for producing the primary apolipoprotein that is present in the central nervous system. *APOE* is a protein consisting of 299 amino acids with a molecular mass of approximately 34 kDa^6^. The enzyme *FTO* (fat mass and obesity-associated), also known as alpha-ketoglutarate-dependent dioxygenase, is encoded by the *FTO* gene located on chromosome 16 in humans^7^. The *LPL* gene, situated on chromosome 8p22, is responsible for the metabolism and transport of lipoproteins in humans^8,9^. However, the exact mechanisms underlying this association remain unclear, and effective prevention of AF in patients with MetS is a clinical challenge. The key pathogenic factor involved in AF development in MetS is yet to be determined. The objective of this study was to determine the expression of *APOE, FTO*, and *LPL* genes in AF patients at the Punjab Institute of Cardiology, Lahore. Additionally, the study aimed to examine the relationship between the expression of these genes and other clinical parameters in AF patients.

## METHODS

This case-control study was conducted in the Department of Zoology, Lahore College for Women University, Lahore. Participants were recruited from the Punjab Institute of Cardiology, Lahore, Pakistan, from July 2021 to June 2022. Subjects were enrolled after providing written informed consent. The study was approved by the Ethical Review Committee (ref. no.: RTPGME-Research-179) of the Punjab Institute of Cardiology and Lahore College for Women University, Lahore, Pakistan.

### RNA isolation and cDNA synthesis

Blood samples were collected for mRNA isolation within 2-4 h of collection, and the Trizol method was used to extract mRNA (Refrigerated Centrifuge Machine HARRIER 18/80, UK). The quality and quantity of mRNA were determined using a Nanodrop (Multiskan SkyHigh Microplate spectrophotometer, UK). The Maxima^®^ First Strand cDNA Synthesis Kit (Thermo Scientific) was used to convert mRNA to cDNA for gene expression (Programmable Thermal Cycler Ptc-06 UK) (Thermo Scientific RevertAid First Strand cDNA Synthesis Kit, cat # K1622). Gel electrophoresis was performed to confirm the cDNA.

### Expression analysis by real-time PCR

To perform real-time PCR (Applied Biosystems Step One^TM^ Real-Time PCR system, Thermo Scientific Fisher Inc., USA), oligonucleotide primers were designed using Primer 3 software. The primer was created by a readily available commercial industry. *APOE* (F: CGGACATGGAGGACGTGT, R: CTGGTACACTGCCAGGCG), *FTO* (F: TGGTGTCCCAAGAAATCGTG, R: TGCAGGCCGTGAACCAC), and *LPL* (F: CCCGAGATGGAGAGCAAAG, R: CCCCTTCCAACTTCCTTCTT) genes’ relative expression was evaluated using the Thermo Scientific Maxima SYBER Green/ROX qPCR Master Mix (CAT # k0221). The Glyceraldehyde 3-phosphate dehydrogenase (GAPDH) gene (F: ATCCCATCACCATCTTCCAGGA, R: CAAATGAGCCCCAGCCTTCT) was used as a reference to normalize the expression of the target gene. One cycle of 94°C for 4 min, followed by 30 cycles of 94°C for 30 s, 59°C for 20-30 s, and 72°C for 45 s, made up the RT-PCR condition. The final extension lasted 5 min at 72°C.

### Statistical analysis

The statistical analysis was carried out using SPSS version 22.0 software. An ANOVA test was utilized to compare mean values among the control group, AF without MetS, and AF with MetS groups. Bivariate Pearson correlation analysis was employed to identify any association between the expression of *APOE, FTO*, and *LPL* genes and the clinical parameters of AF. Stepwise multiple regression was conducted to examine the impact of the expression of *APOE, FTO*, and *LPL* genes on the clinical parameters of AF. The expression of genes was presented as a fold change, and relative gene expression levels were measured using comparative CT (2-ΔΔ^CT^-). A p-value of ≤0.05 was considered significant, whereas a p-value of <0.001 was regarded as highly significant.

## RESULTS

### Demographic and biochemical characteristics of subjects

Table 1 presents the mean±SD values of the studied variables in the control, AF without MetS, and AF with MetS groups.

**Table 1:** Clinical parameters of atrial fibrillation in the control group, atrial fibrillation without the metabolic syndrome group, and atrial fibrillation with the metabolic syndrome group.

Clinical parameters		Control group (n=230)	AF without the MetS group (n=230)	AF with the MetS group (n=230)
Age (years)		57.86±11.32	58.40±11.23	58.40±11.23
Gender, n (%)				
	Male	108 (47)	120 (52)	106 (70)
	Female	122 (53)	110 (48)	124 (54)
SBP (mmHg)		112.43±8.10	126.45±21.42	134.30±36.52**
DBP (mmHg)		85.34±5.80	83.22±13.48	93.04±18.07**
Heart rate (*bpm*)		68.08±6.79	121.45±30.60	116.80±30.83**
BMI (kg/m^2^)		23.37±2.12	29.29±5.55	28.22±10.33**
FBG (mg/dL)		89.60±8.77	122.39±38.56	140.77±50.15**
WHR		0.81±0.05	0.80±0.05	0.93±0.06**
TC (mg/dL)		179.34±18.57	189.29±166.35	187.73±58.73
HDL-C (mg/dL)		56.66±12.53	39.84±23.29	10.68±1.72**
TG (mg/dL)		84.14±66.169	135.01±68.13	156.94±55.99**
LDL-C (mg/dL)		167.88±43.18	185.82±167.82	189.43±74.17
cTnI (ng/mL)		6.40±3.45	0.93±2.19	1.08±3.03**
CK-MB (U/L)		19.49±2.41	55.60±78.81	79.11±84.02**
CPK (U/L)		118.68±28.64	1136.52±4325.53	675.18±3096.93**
Expression of *APOE* gene (arbitrary units)		3.41 fold	0.66 fold	1.59 fold**
Expression of *FTO* gene (arbitrary units)		3.84 fold	1.37 fold	1.14 fold**
Expression of *LPL* gene (arbitrary units)		2.41 fold	0.01 fold	0.24 fold**

p<0.01** is considered a highly significant difference among the groups. SBP: systolic blood pressure; DBP: diastolic blood pressure; bpm: beats per minute; BMI: body mass index; FBG: fasting blood glucose; WHR: waist:hip ratio; TC: total cholesterol; HDL-C: high-density lipoprotein-cholesterol; TG: triglycerides; LDL-C: low-density lipoprotein-cholesterol; cTnI: cardiac troponin I; CK-MB: creatine kinase-MB; CPK: creatine phosphokinase; *APOE*: apolipoprotein E; FTO: fat mass and obesity-associated; *LPL*: lipoprotein lipase.

### Assessment of expression of *APOE, FTO*, and *LPL* genes

The expression profile of the *APOE* gene was decreased by ∼0.66-fold in AF without the MetS group and by ∼1.59-fold in AF with the MetS group as compared to an increase by ∼3.41-fold in the control group, representing a significant difference (Table 1).

The expression profile of the *FTO* gene was decreased by ∼1.37 fold in AF without the MetS group and by ∼1.14-fold in AF with the MetS group as compared to an increase by ∼3.84-fold in the control group, also representing a significant difference (Table 1).

The expression profile of the *LPL* gene was decreased by ∼0.01-fold in AF without the MetS group and by ∼0.24-fold in AF with the MetS group, as compared to an increase by ∼2.41-fold in the control group, representing a significant difference as well (Table 1).

### Pearson correlation analysis

In the AF without MetS group, a highly significant inverse relationship was found between the expression of the *LPL* gene and BMI (body mass index; r=-0.180, p=0.006) and a positive, significant correlation with creatine kinase-MB (CK-MB; r=0.137, p=0.037). In AF with MetS group a significant, inverse relationship was found between the expression of the *FTO* gene with FBG (r=-0.168, p=0.011), the expression of the *LPL* gene (r=-0.163, p=0.013), and a positive relationship with cTnI (r=0.139, p=0.035). In addition, a significant, positive correlation was found between the expression of the *LPL* gene with systolic blood pressure (SBP; r=0.136, p=0.039), HDL-C (r=0.137, p=0.038),a negative association with heart rate (r=-0.307, p=0.001), and the expression of the *FTO* gene (r=-0.163, p=0.013) (Table 2).

**Table 2:** Correlation analysis of gene expression of *APOE, FTO*, and *LPL* genes with the clinical parameters of atrial fibrillation in studied groups.

Clinical parameters	r-value of Exp APOE gene			r-value of Exp FTO gene			r-value of Exp LPL gene		
	Control group	AF without MetS group	AF with MetS group	Control group	AF without MetS group	AF with MetS group	Control group	AF without MetS group	AF with MetS group
Age (years)	-0.004	0.020	0.071	0.072	0.089	-0.015	0.025	0.042	-0.041
SBP (mmHg)	-0.109	-0.073	-0.013	0.043	-0.013	-0.021	-0.092	0.024	0.136*
DBP (mmHg)	-0.029	0.026	-0.022	-0.067	0.033	0.044	-0.029	0.048	-0.038
Heart rate (bpm)	0.092	0.119	0.028	-0.007	-0.025	0.097	-0.125	0.086	-0.307**
BMI (kg/m^2^)	-0.075	-0.005	-0.046	-0.047	0.044	0.015	0.002	-0.180**	0.014
FBG (mg/dL)	0.014	-0.010	-0.056	-0.017	0.007	-0.168*	-0.044	-0.024	0.041
HDL-C (mg/dL)	-0.035	0.033	0.110	0.089	-0.065	0.009	-0.158*	0.047	0.137*
TG (mg/dL)	0.013	0.058	-0.034	0.032	-0.045	0.070	-0.014	0.051	-0.046
cTnI (ng/mL)	-0.252**	0.039	0.106	-0.041	-0.001	0.139*	-0.347**	-0.015	-0.001
CK-MB (U/L)	-0.099	0.028	-0.118	-0.114	-0.089	-0.029	-0.036	0.137*	0.015
CPK (U/L)	-0.102	-0.023	0.001	-0.092	-0.050	-0.039	-0.062	0.118	-0.015
Expression of APOE gene	-	-	-	0.015	0.043	0.004	0.381*	-0.037	-0.108
Expression of FTO gene	0.015	0.043	0.004	-	-	-	-0.020	-0.102	-0.163*
Expression of LPL gene	0.381**	-0.037	-0.108	0.020	-0.102	-0.163*	-	-	-

* Correlation is significant at 0.05 level (two-tailed).

** Correlation is significant at 0.01 level (two-tailed).

### Stepwise regression analysis in the subjects

While for AF without the MetS group, no models were computed with an expression of *APOE* and *FTO* genes as dependent variables, only one model was computed with *LPL* as a dependent variable, where BMI (β=-0.180, p=0.006) was identified as an important predictor of AF. In AF with the MetS group, no model was computed when stepwise multiple regression was employed, considering the expression of the *APOE* gene as a dependent variable. When expression of the *FTO* gene was employed as the dependent variable, three models were computed, indicating FBG (β=-0.168, p=0.011), expression of the *LPL* gene (β=-0.157, p=0.016), and cTn (β=0.153, p=0.018) as important determinants of AF subjects suffering with MetS. Whereas when expression of the *LPL* gene as a dependent variable, three models were computed with heart rate (β=-0.307, p=0.001), expression of the *FTO* gene (β=-0.135, p=0.033), and SBP (β=0.132, p=0.035) as important determinants of AF subjects suffering with MetS (Table 3).

**Table 3:** Stepwise linear regression of atrial fibrillation without and with metabolic syndrome groups.

Variable		B	95% CI		SE B	β	p-value	R^2^	ΔR^2^	Sig. F change
			LL	UL						
AF without MetS group (Expression of LPL gene)										
Model 1								0.033	0.033	0.006
Constant		0.057	0.030	0.083	0.013		0.000			
BMI		-0.001	-0.002	0.000	0.000	-0.180	0.006			
AF with MetS group (Expression of FTO gene)										
Model 1								0.028	0.028	0.011
	Constant	1.941	1.301	2.582	0.325		0.000			
	FBG	-0.006	-0.010	-0.001	0.002	-0.168	0.011			
Model 2								0.053	0.025	0.016
	Constant	2.121	1.470	2.771	0.330		0.000			
	FBG	-0.005	-0.010	-0.001	0.002	-0.162	0.013			
	Exp of *LPL* gene	0.869	-1.576	-0.163	0.359	-0.157	0.016			
Model 3								0.076	0.023	0.018
	Constant	2.086	1.442	2.731	0.327		0.000			
	FBG	-0.006	-0.010	-0.002	0.002	-0.174	0.007			
	Exp of *LPL* gene	-0.866	-1.565	-0.166	0.355	-0.158	0.016			
	cTnI	0.084	0.015	0.154	0.035	0.153	0.018			
(Expression of LPL gene)										
Model 1								0.094	0.094	0.001
	Constant	0.592	0.446	0.739	0.074		0.000			
	Heart rate	-0.003	-0.004	-0.002	0.001	-0.307	0.000			
Model 2								0.112	0.018	0.033
	Constant	0.605	0.459	0.751	0.074		0.000			
	Heart rate	-0.003	-0.004	-0.002	0.001	-0.294	0.000			
	Exp of *FTO* gene	-0.024	-0.047	-0.002	0.011	-0.135	0.033			
Model 3								0.129	0.017	0.035
	Constant	0.458	0.259	0.658	0.101		0.000			
	Heart rate	-0.003	-0.004	-0.002	0.001	-0.293	0.000			
	Exp of *FTO* gene	-0.024	-0.046	-0.002	0.011	-0.132	0.035			
	SBP	0.001	0.000	0.002	0.001	0.132	0.035			

Analysis of data was done using stepwise linear regression. BMI: body mass index; FBG: fasting blood glucose; *FTO*: fat mass and obesity-associated; *LPL*: lipoprotein lipase; cTnI: cardiac troponin I; Exp: expression; SBP: systolic blood pressure.

## DISCUSSION

This study aimed to investigate the expression patterns of metabolic genes (*APOE, FTO*, and *LPL*) in individuals with AF. Results showed that there was a deceased expression of the *APOE, FTO*, and *LPL* genes. This is the first study to report a negative correlation between the expression of the *FTO* gene and FBG, as well as the expression of the *LPL* gene, and a positive correlation between the expression of the *FTO* gene and cardiac troponin 1 in AF subjects with metabolic syndrome. The expression of the *LPL* gene was positively correlated with CK-MB and negatively correlated with BMI in AF subjects. In AF subjects with MetS, the expression of the *LPL* gene was negatively correlated with heart rate and *FTO* gene expression and positively correlated with SBP and HDL-C. The decreased expression of these genes might be influenced by various factors, including environmental and genetic factors.

Our study revealed a positive correlation between the expression of the *LPL* gene and SBP in AF subjects with MetS, as indicated by both correlation and stepwise analysis. This finding is consistent with previous studies that suggest the involvement of the *LPL* gene or nearby genes in BP regulation. For instance, the *LPL* gene and nearby genetic loci have been found to contribute to the variation of BP in the Chinese population. Moreover, the initial association of the *LPL* gene with diastolic blood pressure (DBP) in the Chinese population provides a valuable basis for investigating its role in other populations and races^10^.

In our study, we only used cTnI, CKMB, and CPK as cardiac markers, which have not been reported before. It is also speculated that cardiac markers are linked to the expression of the *APOE* gene, the *FTO* gene, and the *LPL* gene. Cardiac Tn is an intracellular molecule that is involved in heart muscle contraction. Even in healthy individuals, the heart releases small amounts of Tn, but a high concentration of Tn in the blood is a sensitive indicator of myocardial injury^11^. The control group in our study had elevated levels of cTnI, whereas the other groups had decreased levels. Through our correlation analysis and stepwise regression analysis, we discovered a significant association between cTnI and the expression of the *FTO* gene in the group of AF subjects with MetS. Our study investigated the relationship between the expression of the *FTO* gene and cTnI, but we did not provide details on the precise mechanism underlying this association. It is possible that myocardial ischemia due to a rapid heart rate, alterations in microvascular blood flow, inflammation, and fibrosis in both the atrial and ventricular myocardium may play a role^12-16^.

Our findings indicated that the levels of CK-MB were higher in the group of individuals with AF and MetS compared to those with AF subjects and the control group. The results of our correlation analysis revealed a positive association between the expression of the *LPL* gene and CK-MB levels in individuals with AF. Currently, there is no available data on the mechanism underlying the link between the expression of the *LPL* gene and CK-MB.

Our findings showed a decreased expression of the *APOE* gene in both AF subjects and those with metabolic syndrome compared to the control group. This study is the first to report on the expression of the *APOE* gene in atrial fibrillation. However, the specific mechanism of the gene's involvement in AF has not yet been determined. Previous studies have suggested that *APOE* polymorphisms may affect the occurrence of AF and that the *APOE*4 phenotype may be sensitive to AF^17^.

Our study showed a reduction in the expression of the *FTO* gene in both AF with MetS and without MetS groups when compared to the control group. However, it is still unclear how diabetes and obesity impact *FTO* gene expression in the liver. Studies have shown conflicting results, with some suggesting that obese mice with high blood sugar and insulin levels have lower levels of *FTO* mRNA in the liver due to the harmful effects of these conditions^18,19^. Carnevali et al. have concluded that a lack of *FTO* in mice results in an imbalance of autonomic neural modulation of the heart's function towards a sympathetic direction, and it could lead to potentially proarrhythmic remodeling of the heart's electrical and structural properties^20^.

A present study found decreased expression of the *LPL* gene in AF with the MetS group and AF without MetS as compared to a control group. Overall, these studies suggest that the mechanisms behind decreased *LPL* gene expression in humans are complex and might be involved in multiple regulatory pathways, including transcriptional regulation, epigenetic modifications, and post-transcriptional regulation. *LPL* gene expression can also be regulated at the post-transcriptional level through various mechanisms such as mRNA stability, splicing, and translation efficiency. For example, certain microRNAs might inhibit *LPL* expression by targeting the *LPL* mRNA for degradation^21^.

The current study has some limitations, as it only focuses on a few metabolic genes and does not explore the potential mechanisms underlying the association between the expression of *APOE, FTO*, and *LPL* genes and AF in subjects with MetS. Thus, further studies are needed to better understand the molecular mechanisms that underlie the relationship between these genes and AF in patients with MetS.

## CONCLUSION

This study concludes that there is decreased expression of the *APOE, FTO*, and *LPL* genes in AF subjects and AF subjects suffering from MetS as compared to the control group. The decreased expression of these genes might be influenced by various factors, including environmental and genetic factors. Decreased expression of the *APOE, FTO*, and *LPL* genes can have a significant impact on health and disease risk, and further research is needed to fully understand the mechanisms behind these associations. It is suggested that therapeutic intervention targeting the genetic and molecular mechanisms of atrial arrhythmia might help prevent cardiovascular events by reducing the incidence of atrial arrhythmias and their associated complications.

## ETHICAL APPROVAL

The study was conducted according to the guidelines of the Declaration of Helsinki and approved by the Institutional Ethical Review Committee of Lahore College for Women University, Lahore, and was approved by the ethical review committee (ref. no.: RTPGME-Research-179) of Punjab Institute of Cardiology, Lahore, Pakistan.

## CONSENT TO PARTICIPATE

Prior to their participation, all of the participants gave their informed consent, and the data were either pseudo-anonymized or anonymized, depending on the circumstance.
